# CMTM3 Suppresses Proliferation and Osteogenic Transdifferentiation of C2C12 Myoblasts through p53 Upregulation

**DOI:** 10.3390/cells13161352

**Published:** 2024-08-14

**Authors:** Enzhao Shen, Meiyu Piao, Yuankuan Li, Yuecheng Wu, Sihang Li, Sung Ho Lee, Litai Jin, Kwang Youl Lee

**Affiliations:** 1 College of Pharmacy, Research Institute of Pharmaceutical Sciences, Chonnam National University, Gwangju 61186, Republic of Korea; sez2100501005@wmu.edu.cn (E.S.); my101park@gmail.com (M.P.); lyk208943@jnu.ac.kr (Y.L.); 2 School of Pharmaceutical Science, Wenzhou Medical University, Wenzhou 325000, China; wyc221005077@wmu.edu.cn (Y.W.); lsh221005042@wmu.edu.cn (S.L.)

**Keywords:** CMTM3, p53, myoblast, proliferation, transdifferentiation, osteoblast

## Abstract

CKLF-like MARVEL transmembrane domain-containing 3 (CMTM3), a member of the CMTM family that is closely related to tumor occurrence and progression, plays crucial roles in the immune system, cardiovascular system, and male reproductive system. Recently, CMTM3 has emerged as a potential target for treating diseases related to bone formation. However, additional studies are needed to understand the mechanisms by which CMTM3 regulates the process of osteogenic differentiation. In this study, we observed a significant downregulation of *Cmtm3* expression during the transdifferentiation of C2C12 myoblasts into osteoblasts induced by BMP4. *Cmtm3* overexpression suppressed proliferation and osteogenic differentiation in BMP4-induced C2C12 cells, whereas its knockdown conversely facilitated the process. Mechanistically, *Cmtm3* overexpression upregulated both the protein and mRNA levels of p53 and p21. Conversely, *Cmtm3* knockdown exerted the opposite effects. Additionally, we found that Cmtm3 interacts with p53 and increases protein stability by inhibiting proteasome-mediated ubiquitination and degradation. Notably, *Trp53* downregulation abrogated the inhibitory effect of Cmtm3 on BMP4-induced proliferation and osteogenic differentiation of C2C12 myoblasts. Collectively, our findings provide key insights into the role of CMTM3 in regulating myoblast proliferation and transdifferentiation into osteoblasts, highlighting its significance in osteogenesis research.

## 1. Introduction

The growth of skeletal muscle generally involves the proliferation of myoblasts, their differentiation, fusion to form myotubes, and subsequently the development of mature muscle fibers [1,2]. Myoblasts are mononuclear precursor cells that play an essential role in the development, growth, and repair of skeletal muscle tissue [3,4,5]. Studies have reported that, under certain specific conditions, myoblasts are capable of transdifferentiating into a variety of other cell types, including osteoblasts (cells that form bone) [6,7] and adipocytes (fat cells) [8]. This capacity for transdifferentiation offers new possibilities for tissue repair and regeneration [9]. The C2C12 cell line is a well-established model of murine myoblasts that was originally derived from mouse satellite cells [10]. C2C12 cells not only have a rapid proliferation capacity [11], but also have the potential for transdifferentiation [6,7].

Bone is a metabolically active organ that undertakes continuous remodeling during an organism’s lifetime [12,13]. However, imbalances in osteoclast and osteoblast activity can lead to bone diseases, such as osteoporosis [14]. Development of therapeutic strategies that target osteoblast differentiation with minimal side effects is paramount for the effective management of osteogenic diseases. To date, therapeutic options remain limited [15,16,17]. The capacity of other cell types, like myoblasts, to transdifferentiate into osteoblasts may have potential therapeutic applications for certain bone diseases, facilitating bone formation by supplying new osteoblasts [6,7]. Recent reports showed that activation of β-catenin enhanced the proliferation and osteogenic differentiation of skeletal muscle satellite cells at the fracture site, thereby accelerating the healing of osteoporotic fractures [18]. Muscle cell-mediated gene therapy in the bone defect can allow expression of the osteogenic proteins and further enhance bone healing [19]. Moreover, studies revealed that immortalized human myoblast cell line was generated for the delivery of therapeutic proteins using microencapsulation [20], which may have potential for the treatment of bone related diseases. Understanding the regulatory mechanisms of myoblast proliferation and the transdifferentiation of myoblasts into osteoblasts is important for developing new therapeutic strategies and effective management of osteogenic diseases.

Transdifferentiation of myoblasts into osteoblasts is tightly regulated by a network of signaling pathways, including the bone morphogenetic protein (*BMP*) [21], which act to activate downstream transcription factors. Among these, runt-related transcription factor 2 (*Runx2*) is a master regulator, as evidenced by deficits in bone formation observed in *Runx2*-deficient mice [22,23]. Osterix, another factor involved in osteoblast commitment and differentiation, induces the expression of osteogenic markers, such as alkaline phosphatase (*ALP*), and osteocalcin (*BGLAP*) [24,25]. These factors collectively influence the osteogenic process, and their activities are interconnected.

The transcription factor p53, encoded by the *TP53* gene, is one of the most commonly mutated genes in human cancer and a major regulator of cellular stress [26]. There is extensive literature reporting that p53 not only plays a role in skeletal muscle generation but also has an important part to play in bone formation. The loss of Piezo1, which is a mechanosensitive cation channel, causes excessive activation of the ROS-dependent p53-p21 signaling pathway, leading to senescence of skeletal muscle stem cells and resulting in the dysfunction of cellular proliferation and differentiation capabilities, ultimately harming the normal repair and regeneration of skeletal muscle [27]. The literature also reported that a tight control of p53 levels in myoblasts regulates the balance between differentiation and return to quiescence [28]. Emerging evidence also suggests that *Trp53* deficiency can enhance the proliferation and differentiation of both MSCs and bone progenitor cells, ultimately promoting bone formation and contributing to increased bone mass and density [29]. Recent studies have also demonstrated that p53 regulates the differentiation of osteoblasts via the involvement of transcription factors RUNX2/Osterix [30].

The CKLF-like MARVEL transmembrane domain-containing (CMTM) family represents a group of genes implicated in human chemotaxis. This family encompasses chemokine-like factor 1 (*CKLF1*) alongside *CMTM1* through *CMTM8* [31,32]. Among these chemokine-like genes, *CMTM3* is located in a cluster on chromosome 16q22 [33] and possesses tumor suppressor properties across various types of malignant tumors [34,35]. Furthermore, CMTM3 plays crucial roles in the immune system, cardiovascular system, and male reproductive system [36,37]. In addition, a recent study demonstrated the role of CMTM3 in bone formation and osteogenic differentiation of MSCs, both in human MSCs and in a *Cmtm3* knockout mouse model, with associated alterations in the expression of the Erk1/2 and RUNX2 pathway [38]. However, whether CMTM3 regulates the myoblast proliferation and transdifferentiation of myoblasts into osteoblasts and the specific molecular mechanism remains to be clarified.

In this study, we observed that *Cmtm3* overexpression significantly inhibited BMP4-induced proliferation and osteogenic differentiation of C2C12 myoblasts. This inhibitory effect on proliferation and osteogenic differentiation is largely attributed to the upregulation of p53 levels. Our findings demonstrate that Cmtm3 plays a critical role in regulating myoblast proliferation and transdifferentiation into osteoblasts, suggesting its potential as a therapeutic target for modulating bone formation.

## 2. Materials and Methods

### 2.1. Cell Culture and Transfection

Mouse C2C12 cell line and human HEK 293 cell line were procured from the American Type Culture Collection (ATCC, Manassas, VA, USA) and maintained in a humidified incubator at 37 °C with 5% CO_2_. The growth medium consisted of DMEM medium supplemented with 10% fetal bovine serum (FBS; Gibco™, Carlsbad, CA, USA) and 1% penicillin-streptomycin (Gibco™). For transient transfection, the polyethyleneimine (PEI, Polysciences Inc, Warrington, PA, USA)-mediated method was employed. Myc-mouse Cmtm3, HA-human p53, and Flag-human Ubiquitin (Ub) expression plasmids were constructed in a CMV promoter-derived mammalian expression vector (pCS4+). For *Cmtm3* and *p53* knockdown, small interfering RNAs (siRNAs) harboring mouse *Cmtm3* and *Trp53* silencing sequences were utilized. The specific silencing sequences are detailed in Table 1.

### 2.2. In Vitro Osteoblast Differentiation and ALP Staining

Based on previous reports demonstrating its effectiveness in inducing osteogenic differentiation in C2C12 myoblasts, we chose to use BMP4 in our study [39]. To induce osteoblast differentiation, C2C12 cells were treated with 100 ng/mL BMP4 in DMEM medium supplemented with 2% FBS and 1% penicillin-streptomycin for 72 h. To visualize ALP activity, cells were washed twice with phosphate-buffered saline (PBS) and subsequently fixed with 4% paraformaldehyde for 15 min at room temperature. Following fixation, the cells were washed twice with PBS and incubated with 1-Step NBT/BCIP substrate solution (Thermo Fisher Scientific, Waltham, MA, USA) in the dark for 10 min for colorimetric detection of ALP activity. The cells were washed twice with distilled water to stop color reaction. After drying, the images were captured using an automated scanner. For quantitative analysis of ALP activity, the stained cells were lysed in isopropanol for 10 min. Subsequently, the absorbance of the extracted solution was measured at 480 nm using a microplate reader.

### 2.3. Western Blotting

The cells were initially washed twice with PBS and then lysed using a cryo-radioimmunoprecipitation lysis buffer (Abcam, Cambridge, UK), which contained a cocktail of protease and phosphatase inhibitors. Subsequently, the lysates were centrifuged at 13,000 rpm and the supernatant was collected for downstream experiments. For western blot analysis, 30 µg of total protein lysate (per lane) was prepared and mixed with loading buffer (Beyotime, Jiangsu, China). Subsequently, the samples were resolved by SDS–PAGE and transferred onto a polyvinylidene difluoride (PVDF) membrane (Immobilon-P, Millipore, Burlington, MA, USA). To minimize non-specific antibody binding, the membrane was blocked with 5% (*w*/*v*) non-fat dry milk (NFDM) in Tris-buffered saline with 0.1% Tween-20 (TBS-T) for 1 h at room temperature. Subsequently, the membrane was incubated with the appropriate primary antibodies: Cmtm3 (Bioss, Beijing, China, bs-8021R; 1 μg/mL), Runx2 (Hubio, ET1612-47; 200 ng/mL), Osterix (Bioss, bs-1110R; 1 μg/mL), ALP (Abcam, Cambridge, UK, ab229126; 1 μg/mL), p53 (Cell Signaling Technology, Danvers, MA, USA, 2524; 1:1000 dilution), p21 (Abclonal, Woburn, MA, USA, A19094; 1:1000 dilution), GAPDH (Hubio, Woburn, MA, USA, ET1601-4; 200 ng/mL), α-Tubulin (Santa Cruz, CA, USA, sc-8035; 1:1000 dilution). Following primary antibody incubation, the samples were further treated with anti-mouse and anti-rabbit horseradish peroxidase (HRP)-conjugated secondary antibodies, followed by detection using an electrochemical luminescence reagent (Millipore). Protein bands were visualized using the Amersham Image 600 system (GE Healthcare Life Sciences, Marlborough, MA, USA).

### 2.4. Co-Immunoprecipitation (Co-IP) and Ubiquitination Assay

HEK 293 cells were transiently transfected with various combinations (HA-p53 alone or HA-p53 along with Myc-Cmtm3) of HA-p53 and Myc-Cmtm3 expression plasmids. After 48 h of incubation, whole-cell lysates were harvested as previously described. Equal amounts of protein lysates from each sample were incubated with appropriate antibodies (anti-Myc) overnight at 4 °C with gentle agitation. Protein A-Sepharose beads (17096303, GE Healthcare Life Sciences) were then added to each sample at a volume of 40 μL and incubated for an additional 2 h at 4 °C. Subsequently, the immunoprecipitated proteins were resolved by SDS–PAGE and visualized by western blotting. For the ubiquitination assay, HEK 293 cells were transiently transfected with the indicated combinations of HA-p53, Myc-Cmtm3, and Flag-Ub expression plasmids for 24 h. To enrich for ubiquitinated proteins, cells were then treated with proteasome inhibitor MG 132 (10 μM) for an additional 16 h. Harvested cell lysates were incubated with anti-HA antibodies overnight at 4 °C with gentle agitation. Subsequently, the immunoprecipitated proteins were captured using protein A-Sepharose beads and visualized by western blotting.

### 2.5. RNA Isolation and Quantitative Real-Time PCR (RT-qPCR)

Total RNA was extracted from C2C12 cells using TRIzol reagent (Invitrogen, Carlsbad, CA, USA) according to the manufacturer’s instructions. The isolated RNA was then reverse-transcribed into cDNA using the GoScript^TM^ reverse transcription system (Promega, Madison, WI, USA) following the manufacturer’s protocol. Quantitative real-time PCR (RT-qPCR) was performed using the SYBR Green PCR Assay Kit (Thermo Fisher Scientific, Waltham, MA, USA) to amplify target genes. The mRNA levels were normalized to glyceraldehde-3-phosphate dehydrogenase (Gapdh) expression. The relative gene expression levels were calculated using the 2^−ΔΔCt^ method. The specific primer sequences (5′-3′) used for PCR are listed in Table 2.

### 2.6. Immunofluorescence

C2C12 cells were fixed in 4% paraformaldehyde for 15 min, followed by three washes with PBS. Subsequently, the cells were permeabilized in 0.5% Triton X-100 for 15 min at room temperature. After another three washes with PBS, the cells were blocked with 5% BSA. For immunofluorescence staining, the cells were first incubated overnight at 4 °C with Ki67 (D3B5) Rabbit monoclonal antibodies (mAb) (Alexa Fluor^®^647 Conjugate) #12075 (Cell Signaling Technology) at a 1:50 dilution and p53 (1C12) Mouse mAb #2524 (Cell Signaling Technology) at a 1:1000 dilution, followed by the appropriate secondary antibodies. Nuclei were then counterstained with DAPI. For EdU incorporation assay, the BeyoClick™ EdU Cell Proliferation Kit with Alexa Fluor 555 (Beyotime) was utilized according to the manufacturer’s instructions. Nuclei were subsequently counterstained with the included Hoechst 33,342 dye for visualization. Finally, images were visualized and captured via confocal microscopy.

### 2.7. Cycloheximide (CHX) Assay

HEK 293 cells were transfected with indicated combinations of HA-*p53* and Myc-*Cmtm3*. After 24 h of incubation, the cells were treated with 40 μg/mL cycloheximide for 0, 2, 4, and 8 h. The harvested cells were subsequently subjected to SDS–PAGE and determined by western blotting.

### 2.8. Statistical Analysis

Data were analyzed using GraphPad Prism version 8.0. Results are presented as mean ± SEM. Pairwise and multiple comparisons between groups were performed using Student’s *t*-test and ANOVA, respectively. Statistical significance was set at *p* < 0.05.

## 3. Results

### 3.1. Cmtm3 Is Significantly Downregulated during the Transdifferentiation of C2C12 Myoblasts into Osteoblasts Induced by BMP4

**Figure 1:**
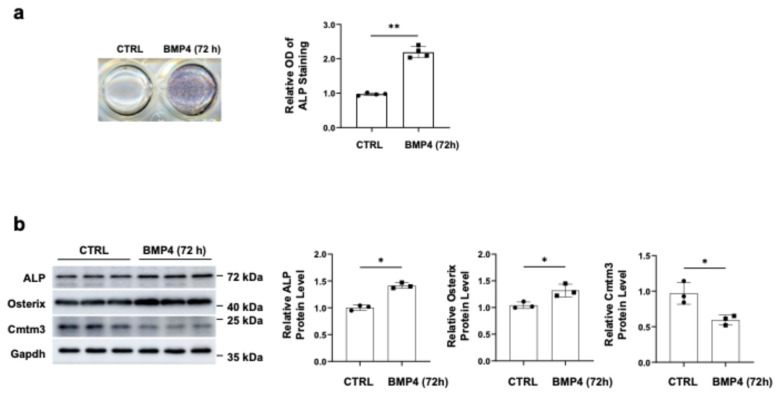
Cmtm3 is significantly downregulated during the transdifferentiation of C2C12 myoblasts into osteoblasts induced by BMP4. C2C12 cells were cultured with BMP4 for 72 h to induce osteoblast differentiation. (**a**) The ALP activity was determined by ALP staining. (**b**) The protein levels of ALP, Osterix, and Cmtm3 were detected by western blotting. Gapdh was used as a loading control. All data are presented as the mean ± standard error of the mean (SEM) of at least three experiments with at least three replicates in each experiment. ** *p* < 0.01 and * *p* < 0.05 compared to the control group (CTRL).

Firstly, we analyzed the expression of *Cmtm3* during the osteogenic differentiation of C2C12 cells. ALP staining revealed a significant upregulation in the osteogenic differentiation ability of C2C12 cells treated with BMP4 for 72 h (Figure 1a). Furthermore, the intracellular protein level of Cmtm3 decreased significantly after BMP4 treatment for 72 h in C2C12 cells, accompanied by increased levels of osteogenic differentiation indicators, such as ALP and Osterix (Figure 1b). Collectively, these findings demonstrate the specific downregulation of intracellular Cmtm3 during the transdifferentiation of C2C12 myoblasts into osteoblasts.

### 3.2. Cmtm3 Overexpression Inhibits Proliferation and Transdifferentiation of Myoblast into Osteoblast in BMP4-Induced C2C12 Cell

**Figure 2:**
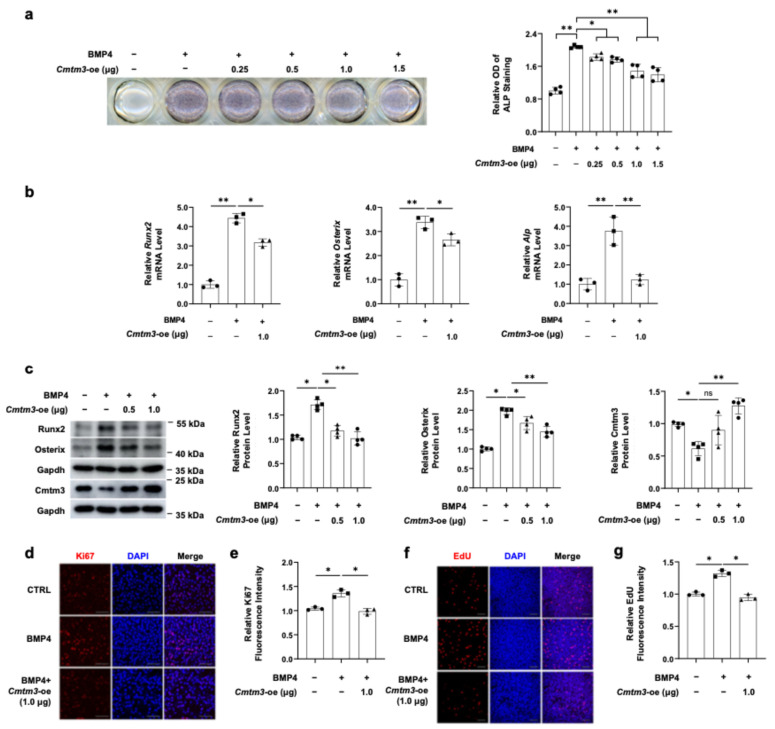
Effect of *Cmtm3* overexpression on myoblast proliferation and transdifferentiation of C2C12 myoblasts into osteoblasts in BMP4-induced C2C12 cells. C2C12 cells were transiently transfected with indicated amount of Myc-*Cmtm3*. The transfected cells were differentiated into osteoblast differentiation with BMP4 treatment for 72 h. (**a**) The ALP activity was determined by ALP staining. (**b**) The mRNA levels of *Runx2*, *Osterix*, and *Alp* were determined by RT-qPCR. (**c**) The protein levels of Runx2, Osterix, and Cmtm3 were detected by western blotting. Gapdh was used as a loading control. (**d**–**g**) Cell proliferation and DNA synthesis were assessed using Ki67 and EdU staining, respectively. Nuclear staining was performed using DAPI or Hoechst dyes. The fluorescence intensities of Ki67 and EdU were quantified and represented graphically. All data are presented as mean ± SEM of at least three experiments with at least three replicates in each experiment. ns, not significant. ** *p* < 0.01 and * *p* < 0.05.

To further clarify the role of CMTM3 in osteogenic differentiation in our model, we utilized plasmid-transfection methods to induce *Cmtm3* overexpression in C2C12 cells. Following successful transfection (Figure 2c), we induced osteogenic differentiation in C2C12 cells using BMP4. ALP staining revealed that *Cmtm3* overexpression inhibited BMP4-induced osteogenic differentiation (Figure 2a). Moreover, *Cmtm3* overexpression significantly decreased the mRNA level of *Runx2*, *Osterix*, and *Alp* during BMP4-induced osteogenic differentiation of C2C12 cells (Figure 2b). Similarly, Western blot analysis demonstrated that *Cmtm3* overexpression led to inhibition of the protein expression of Runx2 and Osterix during BMP4-induced osteoblast differentiation (Figure 2c). These results suggest that *Cmtm3* overexpression inhibits the transdifferentiation of C2C12 myoblasts into osteoblasts. Next, Ki67 and EdU staining experiments were conducted to investigate whether Cmtm3 regulates the C2C12 myoblast proliferation induced by BMP4. Remarkably, *Cmtm3* overexpression significantly decreased proliferation levels in BMP4-treated C2C12 cells (Figure 2d–g). Collectively, these findings suggest that Cmtm3 acts as a negative regulator in myoblast proliferation and transdifferentiation of C2C12 myoblasts into osteoblasts.

### 3.3. Cmtm3 Knockdown Promoted Proliferation and Osteogenic Differentiation in BMP4-Induced C2C12 Cell

**Figure 3:**
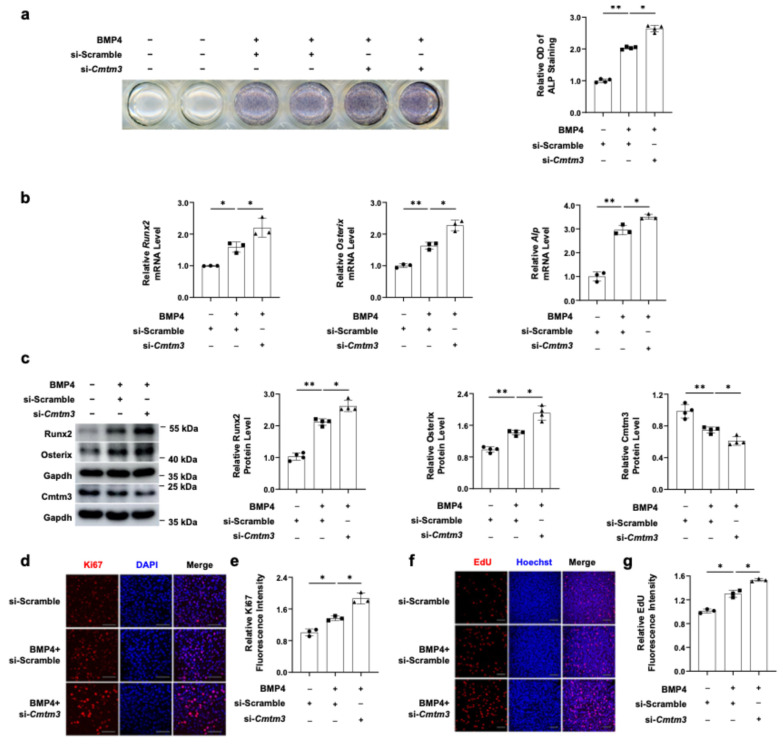
Effect of *Cmtm3* knockdown on myoblast proliferation and transdifferentiation of C2C12 myoblasts into osteoblasts in BMP4-induced C2C12 cells. C2C12 cells were transiently transfected with si-Scramble or si-*Cmtm3*. The transfected cells were differentiated into osteoblast differentiation with BMP4 treatment for 72 h. (**a**) The ALP activity was determined by ALP staining. (**b**) The mRNA levels of *Runx2*, *Osterix*, and *Alp* were determined by RT-qPCR. (**c**) The protein levels of Runx2, Osterix, and Cmtm3 were detected by western blotting. Gapdh was used as a loading control. (**d**–**g**) Cell proliferation and DNA synthesis were assessed using Ki67 and EdU staining, respectively. Nuclear staining was performed using DAPI or Hoechst dyes. The fluorescence intensities of Ki67 and EdU were quantified and represented graphically. All data are presented as the mean ± SEM of at least three experiments with at least three replicates in each experiment. ** *p* < 0.01 and * *p* < 0.05.

To further validate the regulatory role of intracellular Cmtm3 in the process of cell proliferation and osteogenic differentiation, we employed small interfering RNA (siRNA) to knock down *Cmtm3* expression in C2C12 cells. Compared to *Cmtm3* overexpression, ALP staining revealed that *Cmtm3* knockdown promoted BMP4-induced osteoblast differentiation (Figure 3a). Additionally, *Cmtm3* knockdown significantly upregulated the mRNA levels of *Runx2*, *Osterix*, and *Alp* during BMP4-induced osteogenic differentiation (Figure 3b). Furthermore, *Cmtm3* knockdown enhanced the protein expression of Runx2 and Osterix during osteoblast differentiation (Figure 3c). Ki67 and EdU staining experiments were conducted to show that *Cmtm3* knockdown significantly promoted the proliferation of BMP4-induced C2C12 cells (Figure 3d–g). Collectively, these findings suggest that intracellular Cmtm3 plays a crucial role as a regulator of myoblast proliferation and transdifferentiation of C2C12 myoblasts into osteoblasts.

### 3.4. Cmtm3 Overexpression Positively Regulates the Levels of p53 and p21 in BMP4-Treated C2C12 Cells

**Figure 4:**
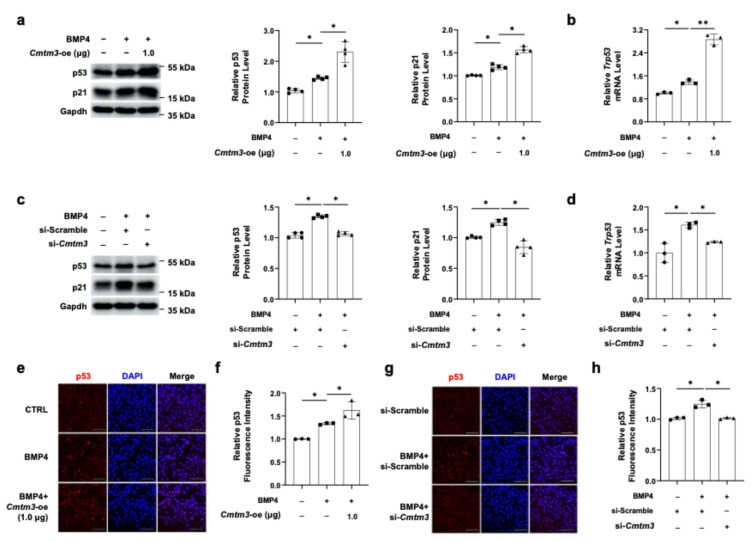
Effect of *Cmtm3* overexpression or knockdown on the expression of p53 and p21 in BMP4-treated C2C12 cells. C2C12 cells were transiently transfected with Myc-Cmtm3 or si-Scramble or si-*Cmtm3*. The transfected cells were treated with BMP4 for 72 h. (**a**,**c**) The protein levels of p53 and p21 were detected by western blotting. Gapdh was used as a loading control. (**b**,**d**) The mRNA levels of *Trp53* were determined by RT-qPCR (**e**–**h**) Nuclear staining was performed using DAPI. The fluorescence intensities of p53 was quantified and represented graphically. All data are presented as mean ± SEM of at least three experiments with at least three replicates in each experiment. * *p* < 0.05 and ** *p* < 0.005 compared to the control group. Scale bar = 100 μm.

Recent research has highlighted the critical roles of both CMTM3 and p53 in tumors, and their relationship has been extensively studied [40,41]. Hence, we hypothesized that the p53 protein might contribute to the regulatory role of Cmtm3 on myoblast proliferation and transdifferentiation of C2C12 myoblasts into osteoblasts. Firstly, we examined the expression of p53 in the absence or presence of Cmtm3. The data revealed that *Cmtm3* overexpression significantly upregulated p53 protein expression during BMP4-induced osteogenic differentiation, as well as its downstream regulatory protein p21, a cyclin-dependent kinase inhibitor (Figure 4a). Consistently, *Cmtm3* overexpression can also significantly upregulate the mRNA levels of *Trp53* during BMP4-induced osteogenic differentiation (Figure 4b). Furthermore, our immunofluorescence result demonstrated that *Cmtm3* overexpression could significantly upregulate p53 expression during BMP4-induced osteogenic differentiation of C2C12 myoblasts (Figure 4e,f). Next, siRNA knockdown experiments were conducted to validate the above-described findings. *Cmtm3* knockdown significantly decreased the protein expression levels of p53 and p21 during BMP4-induced osteogenic differentiation (Figure 4c). Additionally, *Cmtm3* knockdown significantly reduced the mRNA levels of *Trp53* during BMP4-induced osteogenic differentiation in C2C12 cells (Figure 4d). Consistent with these findings, immunofluorescence analyses revealed that *Cmtm3* knockdown can significantly downregulate p53 levels during BMP4-induced osteogenic differentiation in C2C12 cells (Figure 4g,h). In conclusion, *Cmtm3* overexpression positively regulate the levels of p53 and p21 in BMP4-treated C2C12 cells. These results suggest that Cmtm3 regulates myoblast proliferation and transdifferentiation of myoblasts into osteoblasts by upregulating the levels of p53 and p21.

### 3.5. Trp53 Knockdown Abrogates the Inhibitory Effect of Cmtm3 Overexpression on the Cell Proliferation and Osteogenic Differentiation in BMP4-Treated C2C12 Cells

**Figure 5:**
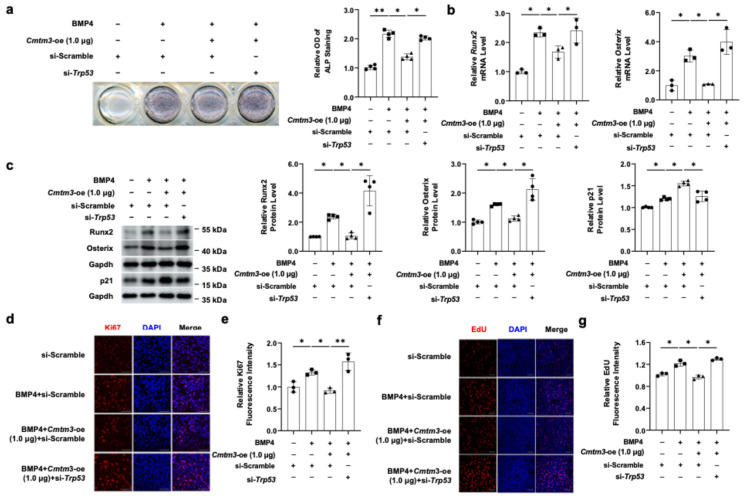
Effect of *Trp53* knockdown on the inhibitory effect of CMTM3 in the cell proliferation and osteogenic differentiation of BMP4-treated C2C12 cells. C2C12 cells were transfected with *Cmtm3* and si-*Trp53* as indicated in combination and treated with BMP4 for 72 h. (**a**) The ALP activity was determined by ALP staining. (**b**) The mRNA levels of *Runx2* and *Osterix* were detected by RT-qPCR. (**c**) The protein levels of Runx2, Osterix, and p21 were detected by western blotting. Gapdh was used as a loading control. (**d**–**g**) Cell proliferation and DNA synthesis were assessed using Ki67 and EdU staining, respectively. Nuclear staining was performed using DAPI or Hoechst dyes. The fluorescence intensities of Ki67, and EdU were quantified and represented graphically. All data are presented as the mean ± SEM of at least three experiments with at least three replicates in each experiment. ** *p* < 0.01 and * *p* < 0.05 compared to the control group. Scale bar = 100 μm.

Next, to further investigate whether the ability of Cmtm3 to inhibit osteogenic differentiation depends on the level of p53, *Trp53* was silenced in C2C12 cells via siRNA (Supplementary Figure S1). ALP staining revealed that *Trp53* knockdown dramatically reversed the inhibitory effect of Cmtm3 on BMP4-induced osteogenic differentiation (Figure 5a). Concurrently, *Trp53* knockdown reversed the downregulation of *Runx2* and *Osterix* mRNA levels induced by *Cmtm3* overexpression (Figure 5b). Furthermore, *Trp53* knockdown effectively reversed the inhibitory effect of *Cmtm3* overexpression on related indicators of osteogenic differentiation, including the downregulation of Runx2 and Osterix protein levels (Figure 5c). These results suggest that the inhibitory effects of *Cmtm3* overexpression on regulating the transdifferentiation of myoblasts into osteoblasts require the involvement of p53. Immunofluorescence techniques with EdU staining and Ki67 staining were used to further confirm whether p53 contributes to the regulatory role of *Cmtm3* overexpression in BMP4-induced C2C12 myoblast proliferation. Consistent with the previous findings, *Cmtm3* overexpression significantly decreased the proliferation level of C2C12 cells induced by BMP4. However, this effect was reversed by *Trp53* knockdown (Figure 5d–g). Collectively, these results demonstrate that *Cmtm3* overexpression can inhibit myoblast proliferation and the transdifferentiation of myoblasts into osteoblasts by p53 upregulation.

### 3.6. CMTM3 Interacts with p53 and Increases Its Protein Stability

**Figure 6:**
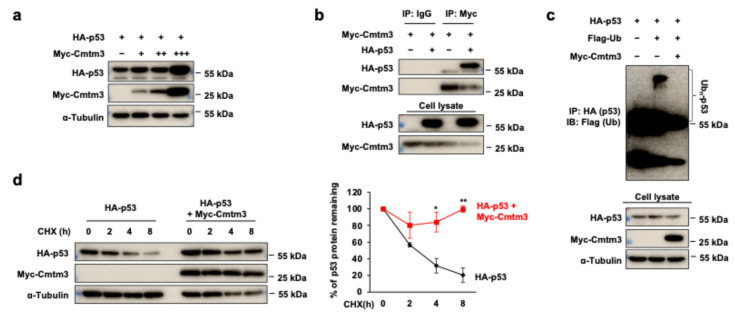
CMTM3-p53 interaction and stability. (**a**) HEK 293 cells were transfected with HA-p53 and increasing amounts (0.125, 0.25, and 0.5 μg) of Myc-Cmtm3. The protein levels of p53 and Cmtm3 were detected by western blotting. α-Tubulin was as a loading control. (**b**) The interaction between p53 and Cmtm3 was examined by co-IP. The anti-Myc (Cmtm3) antibody was used for immunoprecipitation, and the anti-HA (p53) was used for western blotting (top panel). The expression level of the proteins in the lysates was also compared (bottom 2 panels). IgG antibody was used as a control. (**c**) HEK 293 cells were transfected with HA-p53 alone or with Myc-Cmtm3, and then treated with CHX (40 μg/mL) for the indicated periods of time. The protein levels of p53 and Cmtm3 were detected by western blotting. α-Tubulin was used as a loading control. (**d**) HEK 293 cells were transfected with the indicated combinations of Flag-ubiquitin (Ub), Myc-Cmtm3, and HA-p53 plasmids. The ubiquitination levels of p53 were detected by IP with anti-HA (p53) antibody, followed by western blotting with anti-Flag (Ub) antibody (top panel). The level of Cmtm3 and p53 in the cell lysates is shown (bottom panels). α-Tubulin was used as a loading control. All data are presented as the mean ± SEM of at least three experiments with at least three replicates in each experiment. ** *p* < 0.01 and * *p* < 0.05 compared to the control group.

To elucidate the precise relationship between CMTM3 and p53, we transfected HEK 293 cells to overexpress both proteins. Firstly, we observed an increase in exogenous protein levels of p53 upon *Cmtm3* overexpression (Figure 6a). Furthermore, we detected a direct interaction between Cmtm3 and p53 (Figure 6b). Next, CHX and ubiquitination assays were conducted to confirm whether Cmtm3 affects p53 protein stability through direct binding. Our findings demonstrated that Cmtm3 prolonged the protein half-life of p53 (Figure 6c). Moreover, Cmtm3 prevented p53 degradation by inhibiting proteasomal-mediated ubiquitination (Figure 6d). These findings suggest that Cmtm3 enhances the protein stability of p53 by inhibiting its ubiquitination.

## 4. Discussion

In this study, we initially observed a decrease in Cmtm3 expression during the BMP4-induced transdifferentiation of C2C12 myoblasts into osteoblasts. Subsequently, through *Cmtm3* overexpression and silencing experiments, we confirmed that Cmtm3 negatively regulated both BMP4-induced proliferation and osteogenic differentiation in C2C12 myoblasts. Mechanistically, we elucidated that p53 acted as a crucial mediator in the effect of Cmtm3 on cell proliferation and osteogenic differentiation of C2C12 cells. Furthermore, we identified a direct protein–protein interaction between Cmtm3 and p53 and observed that Cmtm3 enhanced p53 protein stability and reduced p53 ubiquitination.

Myoblasts need to go through a phase of proliferation before differentiating into myofibers [2,4]. Myoblast proliferation provides the necessary number of cells for differentiation, which is a prerequisite for the maturation process of muscle cells [1,42]. The proliferation of myoblasts plays a key role in muscle growth, repair, maintenance of function, and response to injury and disease [43,44]. Methods such as the Ki67 and EdU assays are commonly used to measure myoblast proliferation [45,46]. CMTM3 has been reported to be involved in regulating the proliferation levels of many types of cells under various disease condition. The role of CMTM3 in cancer progression remains controversial. While *CMTM3* overexpression has been shown to promote proliferation in pancreatic cancer [34], it can also inhibit human testicular cancer cell growth [41]. These contrasting findings suggest that the specific functions of CMTM3 in tumor development may be context-dependent, varying based on cancer type and the specific microenvironmental conditions. Therefore, we hypothesize that CMTM3 may be involved in regulating the proliferation levels of myoblasts. Subsequently, in line with our hypothesis, using Ki67 and EdU cell proliferation detection technology, we firstly found that *Cmtm3* overexpression was negatively correlated with the BMP4-induced proliferation level of C2C12 cells. Myoblasts have also been found to have the potential to transform into osteoblasts under certain conditions [6,7]. This ability to transdifferentiate opens up new possibilities for tissue engineering and regenerative medicine, contributing to the development of new methods to promote bone tissue repair and regeneration [9]. Numerous transcription factors are involved in the establishment and maintenance of osteoblast phenotypes, including Runx2 and Osterix [47]. Defects in Runx2 are a major cause of cranial dysplasia [48]. Runx2 enhances the proliferation of suture MSCs and induces their commitment to osteoblast lineage cells [49]. Osterix, located downstream of Runx2, not only affects endochondral osteogenesis by participating in the terminal cartilage differentiation but also influences intramembranous bone formation [24,50]. Consistent with previous literature, our study demonstrated that *Cmtm3* overexpression inhibited the transdifferentiation of C2C12 myoblasts into osteoblasts and downregulated the expression of Runx2 and Osterix. Conversely, *Cmtm3* knockdown can significantly upregulate the BMP4-induced transdifferentiation of C2C12 cells into osteoblasts. However, further studies are needed to determine whether Cmtm3 directly regulates myoblast proliferation and the transdifferentiation of C2C12 myoblasts into osteoblasts.

The tumor suppressor p53 plays a complex and critical role in the proliferation of myoblasts. For example, the literature reports that MiR-16-5p targets SESN1 to regulate the p53 signaling pathway, affecting the proliferation of myoblasts [51]. The tumor suppressor p53 also exerts a repressive effect on bone development and remodeling. Osteoblast differentiation is a key component of bone formation, and the relationship between p53 and osteoblast differentiation has been extensively studied. p53 plays a negative role in postnatal bone development and bone formation [52,53,54]. In vivo studies demonstrated that mice lacking *Trp53* exhibit increased bone formation and an osteosclerosis phenotype [29]. Similarly, in vitro studies have shown that osteoblasts lacking p53 exhibit increased proliferation and accelerated differentiation [30]. Both CMTM3 and p53 play important roles in tumor development and osteogenic differentiation, and their relationship has been extensively reported in the literature. For instance, CMTM3 inhibits chordoma cell proliferation by accelerating EGFR degradation and upregulating p53 expression [40]. Additionally, the expression of CMTM3 significantly inhibits the proliferation and migration of testicular cancer cells, accompanied by the activation of *p53* transcription, induction of p53 accumulation, and upregulation of p21 expression [41]. Here, we hypothesized that the p53 protein might be involved in the regulation of CMTM3 on myoblast proliferation and the transdifferentiation of C2C12 myoblasts into osteoblasts. As expected, the *Cmtm3* overexpression significantly upregulated p53 and its downstream regulatory protein p21 levels during the BMP4-inudced transdifferentiation of C2C12 myoblasts into osteoblasts. Conversely, *Cmtm3* knockdown downregulated p53 and p21 levels during osteogenic differentiation. To this end, *Trp53* knockdown could reverse the inhibitory effect of *Cmtm3* overexpression on the BMP4-induced proliferation and osteogenic differentiation of C2C12 cells. Based on these findings, we concluded that the ability of CMTM3 to inhibit the proliferation and osteogenic differentiation in BMP4-induced C2C12 cells is largely dependent on p53 upregulation.

Post-translational modification of p53, including ubiquitination, phosphorylation, and acetylation, is among the key mechanisms regulating the activity of p53 [55]. p53 is usually activated in a phosphorylated or acetylated form and deactivated in a ubiquitinated or ubiquitin-like form [56]. High levels of ubiquitination directly mediate the degradation of p53 by the proteasome. The ubiquitin–proteasome pathway plays an important role in regulating p53 protein levels [57]. We first revealed through co-IP experiments that there is a direct interaction between Cmtm3 and p53 protein. Using CHX assays, we demonstrated that Cmtm3 increases the protein stability of p53, thereby increasing its half-life. In addition, we proposed that the upregulation of p53 protein stability by *Cmtm3* overexpression, along with reduced poly-ubiquitination, suggests a potential impact via degradation mediated by the ubiquitin–proteasome system. Further research is needed to investigate the possibility that other post-translational modifications, such as phosphorylation or acetylation, are involved in this regulatory process. Additionally, studies are needed to investigate the possibility that E3 ligases, such as MDM-2, are involved in the regulation of p53 ubiquitination.

While our research elucidated the interaction between p53 and CMTM3, further investigation is warranted to explore the potential involvement of other signaling pathways in CMTM3’s regulation of myoblast proliferation and transdifferentiation into osteoblasts. Notably, our study did not address the potential contribution of pathways such as Erk1/2, which have been implicated in CMTM3-mediated control of cell proliferation and osteogenic differentiation in previous studies [38]. Additionally, the potential role of CMTM3 in modulating myoblast differentiation into myotubes remains to be elucidated.

## 5. Conclusions

In conclusion, our study is the first to identify that CMTM3 plays a negative regulatory role in myoblast proliferation and the BMP4-induced transdifferentiation of myoblasts into osteoblasts. Furthermore, we found that increased p53 expression is a critical mediator of this CMTM3 function. Moreover, we provided evidence for a direct interaction between CMTM3 and p53, as well as for the regulation of ubiquitination on protein stability. Taken together, our results suggest a pivotal role for CMTM3 in regulating myoblast proliferation and the transdifferentiation of myoblasts into osteoblasts. These findings pave the way for research on therapeutic targets for bone metabolic diseases, offering potential avenues for novel treatments.

## Figures and Tables

**Table 1:** The target sequences for siRNAs.

siRNA		Sequence
si-*Cmtm3*	Forward	GCC GUU UAC UUC CUC UUU GCU TT
	Reverse	AGC AAA GAG GAA GUA AAC GGC TT
si-*Trp53*	Forward	ACU ACA AGU ACA UGU GUA AUA TT
	Reverse	UAU UAC ACA UGU ACU UGU AGU TT

**Table 2:** The primer sequences for RT-qPCR.

Gene Name		Sequence
*Runx2*	Forward	CCT GAA CTC TGC ACC AAG TCC T
	Reverse	TCA TCT GGC TCA GAT AGG AGG G
*Osterix*	Forward	TCG CAT CTG AAA GCC CAC TT
	Reverse	CTC AAG TGG TCG CTT CTG GT
*Alp*	Forward	ATC TTT GGT CTG GCT CCC ATG
	Reverse	TTT CCC GTT CAC CGT CCA C
*Trp53*	Forward	CTT CCT CCA GAA GAT ATC CTG
	Reverse	GCC ATA GTT GCC CTG GTA AG
*Gapdh*	Forward	ACT TGA AGG GTG GAG CCA AA
	Reverse	GAC TGT GGT CAT GAG CCC TT

## Data Availability

The data presented in this study are available in the article.

## Supplementary Materials

The following supporting information can be downloaded at: https://www.mdpi.com/article/10.3390/cells13161352/s1, Figure S1:si-p53 knockdown validation.
