# Supplementary material for: CMTM3 Suppresses Proliferation and Osteogenic Transdifferentiation of C2C12 Myoblasts through p53 Upregulation
Source: Cells. 2024 Aug 14;13(16):1352. doi: 10.3390/cells13161352 (PMC11352514; doi:10.3390/cells13161352)
Supplement: Supplementary file 1 [file cells-13-01352-s001.zip › cells-3136908-supplementary.pdf]

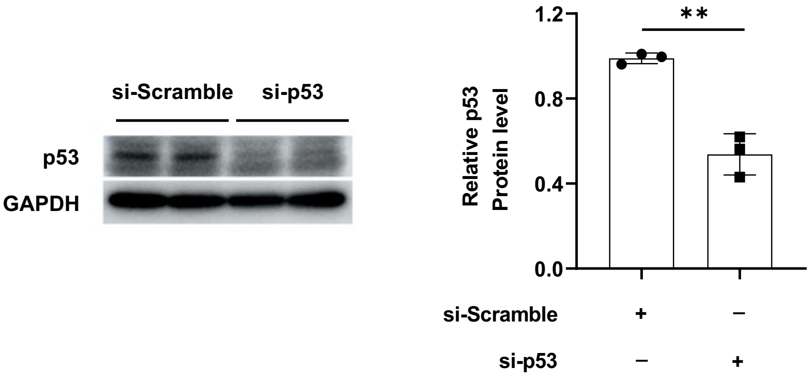

Supplementary Figure S1. si-p53 knockdown validation. The protein levels of p53 were compared between control or p53 knockdown C2C12 cells at 48 h post-si-p53 transfection. GAPDH was used as a loading control. All data are presented as the mean ± SEM of at least three experiments with at least three replicates in each experiment. ** $p < 0.01$ compared.
